# RNA sequencing provides exquisite insight into the manipulation of the alveolar macrophage by tubercle bacilli

**DOI:** 10.1038/srep13629

**Published:** 2015-09-08

**Authors:** Nicolas C. Nalpas, David A. Magee, Kevin M. Conlon, John A. Browne, Claire Healy, Kirsten E. McLoughlin, Kévin Rue-Albrecht, Paul A. McGettigan, Kate E. Killick, Eamonn Gormley, Stephen V. Gordon, David E. MacHugh

**Affiliations:** 1 Animal Genomics Laboratory, UCD School of Agriculture and Food Science, University College Dublin, Belfield, Dublin 4, Ireland; 2 UCD School of Veterinary Medicine, University College Dublin, Belfield, Dublin 4, Ireland; 3 Systems Biology Ireland, UCD Conway Institute of Biomolecular and Biomedical Research, University College Dublin, Dublin 4, Ireland; 4 Tuberculosis Diagnostics and Immunology Research Centre, UCD School of Veterinary Medicine, University College Dublin, Belfield, Dublin 4, Ireland; 5 UCD School of Medicine and Medical Science, University College Dublin, Belfield, Dublin 4, Ireland; 6 UCD School of Biomolecular and Biomedical Science, University College Dublin, Belfield, Dublin 4, Ireland; 7 UCD Conway Institute of Biomolecular and Biomedical Research, University College Dublin, Dublin 4, Ireland

## Abstract

*Mycobacterium bovis*, the agent of bovine tuberculosis, causes an estimated $3 billion annual losses to global agriculture due, in part, to the limitations of current diagnostics. Development of next-generation diagnostics requires a greater understanding of the interaction between the pathogen and the bovine host. Therefore, to explore the early response of the alveolar macrophage to infection, we report the first application of RNA-sequencing to define, in exquisite detail, the transcriptomes of *M. bovis*-infected and non-infected alveolar macrophages from ten calves at 2, 6, 24 and 48 hours post-infection. Differentially expressed sense genes were detected at these time points that revealed enrichment of innate immune signalling functions, and transcriptional suppression of host defence mechanisms (*e.g*., lysosome maturation). We also detected differentially expressed natural antisense transcripts, which may play a role in subverting innate immune mechanisms following infection. Furthermore, we report differential expression of novel bovine genes, some of which have immune-related functions based on orthology with human proteins. This is the first in-depth transcriptomics investigation of the alveolar macrophage response to the early stages of *M. bovis* infection and reveals complex patterns of gene expression and regulation that underlie the immunomodulatory mechanisms used by *M. bovis* to evade host defence mechanisms.

Bovine tuberculosis is a chronic infectious disease of domestic livestock and wildlife caused by infection with *Mycobacterium bovis*, a pathogenic mycobacterial species belonging to the *Mycobacterium tuberculosis* complex (MTBC)^1^. As a zoonotic agent, *M. bovis* infection also has serious implications for human health^2^. Previous studies have shown that the aetiology and host immune response to *M. bovis* infection in cattle is nearly identical to *M. tuberculosis* infection in humans^3^,^4^,^5^.

*M. bovis* is primarily transmitted via inhalation of aerosolized bacteria, with the primary site of infection being the lung^6^. Following inhalation, the pathogen is phagocytosed by host alveolar macrophages, which serve as key effector innate immune cells and usually can kill intracellular bacilli or contain infection via the activity of inflammatory cytokines^7^. *M. bovis*, however, can persist and replicate within alveolar macrophages via diverse mechanisms that subvert or exploit host immune responses^8^. These mechanisms include prevention of macrophage phagosome-lysosome fusion, inhibition of apoptosis, suppression of antigen presentation and signalling mechanisms within the macrophage, and induction of necrosis, culminating in immunopathology and ultimately shedding of the pathogen from the host^9^,^10^,^11^,^12^.

We hypothesised that mechanisms used by pathogenic mycobacteria to overcome innate immunity and establish infection would be revealed through analysis of the gene expression changes in the macrophage response to infection. Previous transcriptomics studies of the bovine macrophage response to *M. bovis* infection, performed by us and others^13^,^14^,^15^, have yielded important insights into the molecular mechanisms and cellular pathways that govern mycobacteria-macrophage interplay. Here, we have extended this earlier work by performing the first in-depth analysis of the primary innate immune cell, the alveolar macrophage, response to infection with *M. bovis* using high-throughput RNA-sequencing (RNA-seq). We have analysed gene expression changes in purified bovine alveolar macrophages from ten age-matched unrelated Holstein-Friesian male calves infected with *M. bovis* across a 48 h time course.

The work described here provides important new information regarding mammalian host responses to mycobacterial pathogens for the following reasons. Firstly, we have used *M. bovis* to infect bovine alveolar macrophages–the primary host cell type that encounters *M. bovis* following inhalation^16^,^17^; earlier studies have generally used blood-derived macrophages^13^,^14^,^15^,^18^. Secondly, culture of *M. bovis* was performed in the absence of the detergent Tween 80 as previous research has shown that Tween 80 alters the cell wall composition and morphology of mycobacteria which may affect the interaction of mycobacteria with macrophages during *in vitro* infection studies^19^,^20^,^21^,^22^. Thirdly, to the best of our knowledge, all previous investigations of the alveolar macrophage transcriptome in infection with tubercle bacilli have involved the analysis of microarray data; none have used RNA-seq, which offers unprecedented opportunities for global gene expression analysis, including unbiased whole-transcriptome gene expression profiling, cataloguing of sense and antisense transcription, and discovery of novel RNA transcripts^23^,^24^.

## Materials and Methods

The laboratory methods have previously been described by us^25^ and therefore a summary is provided below. Detailed materials and methods are provided in Supplementary Methods and the complete bioinformatics pipeline is accessible online^26^.

### Ethics Statement

All animal procedures were performed according to the provisions of the Cruelty to Animals Act of 1876 and ethical approval was obtained from the University College Dublin Animal Ethics Committee (protocol number AREC-13-14-Gordon).

### Animal work, lung lavages and alveolar macrophage purification

Fourteen unrelated, age-matched Holstein-Friesian male calves were selected from a tuberculosis-free herd and total lung cells were harvested by pulmonary lung lavage via tracheal infusion of calcium- and magnesium-free Hank’s Balanced Salt Solution (HBSS; Invitrogen^™^, Life Technologies Corp., Paisley, UK). All animals tested negative after microbial screening. The HBSS-cell suspension was washed by centrifugation, resuspended in foetal bovine serum (FBS) with 10% dimethyl sulfoxide (Sigma-Aldrich Ltd.), aliquoted and stored at −140 °C using Mr. Frosty^®^ Cryo 1 °C Freezing Containers (Nalgene^®^, Thermo Fisher Scientific, Waltham, MA, USA).

Approximately 1.5 × 10^8^ total lung cells from each animal were thawed in a 37 °C water bath for 1 min and transferred into R10^+^ media (RPMI 1640 medium [Invitrogen^™^] supplemented with 10% FBS, 2.5 μg/ml amphotericin B, 2 mM L-glutamine, 100 μg/ml ampicillin and 25 μg/ml gentamycin [all from Sigma-Aldrich Ltd.]). Cells were centrifuged, resuspended in R10^+^ media, placed in a vented culture flask (CELLSTAR^®^, Greiner Bio-One Ltd., Stonehouse, UK) and incubated for 24 h at 37 °C, 5% CO_2_. After incubation, media was removed and adherent cells were dissociated by adding 1× non-enzymatic cell dissociation solution (Sigma-Aldrich Ltd.). Cells were centrifuged, washed, seeded at 5 × 10^5^ viable cells/well in culture plates (Sarstedt Ltd.) and incubated for 24 h at 37 °C, 5% CO_2_. The purity of the seeded macrophages was confirmed by flow cytometry with anti-CD14 antibody.

### Culture of *M. bovis*

*M. bovis* AF2122/97 strain was cultured to late logarithmic phase in Middlebrook 7H9 media (Difco^™^, Becton, Dickinson Ltd., Oxford, UK) enriched with 1× Middlebrook albumin-dextrose-catalase (ADC; Difco^™^) and 10 mM final concentration of sodium pyruvate (Sigma-Aldrich Ltd.) and then stored at −80 °C. Prior to infection, 1 ml *M. bovis* culture stock was thawed and cultured until mid-logarithmic phase. Further culturing was performed statically in vented Corning^™^ Erlenmeyer flasks (Thermo Fisher Scientific Inc.) until late-logarithmic phase. On the day of infection, the *M. bovis* culture was centrifuged, resuspended in R10^−^ media (R10^+^ media without antibiotics) and sonicated for 1 min. The cell number was calculated from OD_600nm_ and adjusted to 5 × 10^6^ bacterial cells/ml using R10^−^ media.

### *M. bovis*-infection of alveolar macrophages and macrophage RNA extraction

For infections, the media from all wells containing alveolar macrophages was removed and replaced with 1 ml R10^−^ media containing *M. bovis*, yielding a multiplicity of infection of 10 bacilli per macrophage. Parallel non-infected control alveolar macrophage received 1 ml R10^−^ media only. Alveolar macrophages were then incubated at 37 °C, 5% CO_2_ for 2, 6, 24 and 48 h. After 2 h post-infection (hpi), the media from all infection experiments was replaced with fresh R10^−^ media and culture plates were reincubated at 37 °C, 5% CO_2_ until cells were required for harvesting. Alveolar macrophages were lysed by adding 250 μl/well RLT buffer supplemented with 1% β-mercaptoethanol (Qiagen Ltd., Crawley, UK) and stored at −80 °C until required for RNA extraction.

All RNA extractions were performed using an RNeasy^®^ Plus Mini kit (Qiagen Ltd.) according to the manufacturer’s instructions. RNA quantity and quality was assessed and all samples displayed a 260/280 ratio greater than 2.0 and RNA integrity numbers greater than 8.5. RNA samples were stored at −80 °C.

### Strand-specific RNA-seq library preparation and sequencing

The samples used for RNA-seq library preparation comprised *M. bovis*-infected and non-infected samples from each post-infection time points across 10 animals (except for one animal that did not yield sufficient macrophages for the 48 hpi time point).

Approximately 200 ng of total RNA from each sample was used to prepare individually barcoded strand-specific RNA-seq libraries. Two rounds of poly(A)+ RNA purification were performed for all RNA samples using the Dynabeads^®^ mRNA DIRECT^™^ Micro Kit (Invitrogen^™^) according to the manufacturer’s instructions. The poly(A) + RNA was then used to generate strand-specific RNA-seq libraries using the ScriptSeq^™^ v2 RNA-Seq Library Preparation Kit, the ScriptSeq^™^ Index PCR Primers (Sets 1 to 4) and the FailSafe^™^ PCR enzyme system (all from Epicentre^®^, Illumina^®^ Inc., Madison, WI, USA) according to the manufacturer’s instructions. RNA-seq libraries were purified using the Agencourt^®^ AMPure^®^ XP system (Beckman Coulter Genomics, Danvers, MA, USA) according to the manufacturer’s instructions for double size selection (0.75× followed by 1.0 × ratio). Individually barcoded RNA-seq libraries were pooled in equimolar quantities and assessed for quantity and quality.

Cluster generation and paired-end 2 × 90 nucleotide read sequencing of the pooled RNA-seq libraries were performed by BGI (BGI–Hong Kong, Hong Kong, China) using an Illumina^®^ HiSeq^™^ 2000 sequencer. All RNA-seq data are accessible from the NCBI GEO database^27^ (accession number GSE62506).

### Bioinformatics and differential expression analyses of RNA-seq data

Computational analyses consisted of an initial quality check of the raw reads data files using the FastQC software^28^. Subsequently, a Perl script was used to deconvolute barcoded reads into individual libraries, filter out adapter sequence reads, and remove poor quality reads. Paired-end reads from individual libraries were then aligned to the *B. taurus* reference genome (UMD3.1.71) using the STAR aligner software^29^.

For each library, uniquely aligned paired-end reads were used to obtain raw counts for all bovine genes (*B. taurus* UMD3.1.71 Ensembl genome annotation^30^) based on sense strand data with the featureCounts software^31^. Subsequently, the gene counts were used for differential expression analysis with the edgeR package^32^ and the following steps: lowly expressed gene filtering, normalisation using the trimmed mean of M-values method, estimation of dispersion using the Cox-Reid method, evaluation of differential gene expression between *M. bovis*-infected versus non-infected samples at each time point (*i.e*., paired-sample statistical model) using a negative binomial generalised linear model, and correction for multiple testing using the Benjamini-Hochberg method^33^.

For antisense gene analyses, the uniquely aligned paired-end reads that were unassigned to any sense genes were used to generate raw counts for antisense genes using the featureCounts software. For this, the *B. taurus* UMD3.1.71 genome annotation was configured to include a promoter and terminator region^34^ and to exclude proximal genes. In addition, to remove antisense gene artefacts^35^, we modified the method of Perocchi and colleagues^36^. This involved generation of additional raw counts with featureCounts, a low gene expression filtering step and the computation of count ratios as shown below.
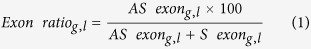

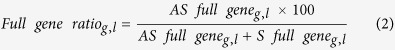
*AS* = antisense count; *S* = sense count; *g* = gene; *l* = library.

A greater-sided pairwise Wilcoxon signed-rank test was then used to assess significant differences between the full gene ratio and the exon ratio for each gene. Antisense genes found to be statistically significant for this ratio filtering step were then used for differential antisense gene expression analysis with the edgeR package as described for the sense genes.

For novel genes analyses, aligned reads were processed with the Cufflinks software^37^ using the reference annotation-based transcript assembly method^38^ and the *B. taurus* reference genome annotation (UMD3.1.71) to generate a *de novo* transcripts assembly. The reciprocal best hit (RBH) method^39^ was then used to annotate putative novel genes by orthology to *Homo sapiens* reference protein sequences (*H. sapiens* GRCh37.73) using standalone BLAST^40^ and Perl scripts. Subsequently, the uniquely aligned paired-end reads not assigned to any sense or antisense gene were used to compute raw counts for novel genes using the featureCounts software and the *de novo B. taurus* genome annotation. Then raw counts for each novel gene were used for differential expression analysis with the edgeR package as described for the sense and antisense genes.

### Systems analyses

Using the biomaRt package^41^, each *B. taurus* sense gene was annotated with its *H. sapiens* ortholog from Ensembl (GRCh37.71) to facilitate integration with the differentially expressed (DE) novel genes data set and to provide consistent data input across the systems biology analysis tools. The Ingenuity^®^ Systems Pathway Analysis (IPA) software package (Qiagen Corp., Redwood City, CA, USA) was used to identify over-represented canonical pathways based on the Ingenuity^®^ Knowledge Base. To further focus on the canonical pathways of most relevance to our study, the Pathway–Guide software package (Advaita Corp., Plymouth, MI, USA) was also used to perform signalling pathway impact analysis^42^; this method uses biological interaction data from the KEGG^43^ and Reactome^44^ databases. Finally, the Sigora package^45^ was used to perform signature over-representation analysis based on the KEGG and Reactome databases.

### cDNA synthesis and qPCR analysis

For technical validation, cDNA was prepared from the same RNA used for the RNA-seq library preparations; for parallel biological validation, cDNA was prepared from RNA extracted from macrophage samples purified from four additional calves. The High Capacity cDNA Reverse Transcription Kit (Applied Biosystems^®^, Life Technologies Corp., Warrington, UK) was used to prepare cDNA from 60 ng total RNA according to the manufacturer’s instructions. Quantitative real-time PCR (qPCR) was performed using Fast SYBR^®^ Green Master Mix (Applied Biosystems^®^) on a 7500 Fast Real-Time PCR System (Applied Biosystems^®^) according to the manufacturer’s instructions. Reactions contained 3 μl diluted cDNA samples (or appropriate controls), 10 μl of SYBR mix and 300 nM final concentration of each primer (Supplementary Data S1, worksheet 1). PCR cycling conditions comprised a 50 °C step for 2 min, a 95 °C step for 20 s, followed by 40 cycles at 95 °C for 3 s and 60 °C for 30 s.

The qbase^+^ software tool (Biogazelle NV, Zwijnaarde, Belgium)^46^ was used for qPCR normalisation (the GeNorm algorithm^47^ identified *PPIA* and *H3F3A* as suitable reference genes). Gene expression fold-changes were computed for *M. bovis*-infected versus non-infected samples at each time point using calibrated normalised relative quantities. Normal distribution of fold-change values was checked with the Shapiro-Wilk test in the SPSS statistical package (IBM Corp., Armonk, NY, USA). Two-tailed paired sample *t*-tests and pairwise Wilcoxon signed-rank tests were used to assess statistically significant gene expression fold-changes for normally and non-normally distributed data, respectively. The Pearson correlation (*r*) between RT-qPCR and RNA-seq gene expression fold-change was estimated.

## Results and Discussion

### RNA-seq summary statistics

In total, 78 individually barcoded strand-specific RNA-seq libraries (*i.e*., *M. bovis*-infected and non-infected macrophages from ten animals at 2, 6 and 24 hpi and nine animals at 48 hpi) were sequenced on an Illumina^®^ HiSeq^™^ 2000 apparatus, generating a total of 2.2 billion paired-end (2 × 90 nucleotides) reads. Deconvolution and filtering of sequence reads yielded a mean of 22.7 million paired-end reads per individual library (Supplementary Data S2). These data satisfy previously defined criteria for RNA-seq experiments in terms of the number of independent biological replicates per treatment and the sequencing depth^48^,^49^. Alignment of the filtered paired-end reads to the *B. taurus* reference genome yielded mean values per library of 20.4 million reads (89.72%) mapping to unique locations. Gene count summarisation revealed mean values per library of 14.9 million reads (73.32%) assigned to Ensembl gene IDs based on sense strand sequence information, with 0.9 million reads (4.34%) assigned to gene IDs based on antisense strand sequence information and 0.5 million reads (2.27%) assigned to gene IDs based on novel gene annotation analysis. These three sets of sequence reads were used to separately derive gene expression values for sense gene data, antisense gene data and novel gene data, respectively.

### Differential gene expression analyses

A stringent gene expression filtering criterion was applied to the sense strand expression data to remove lowly expressed genes and thereby reduce Type I error^50^. This yielded 11,928 sense genes (48.46% of total *B. taurus* reference genes) that were suitable for differential expression analysis. The data from all 11,928 filtered sense genes were used for multidimensional scaling analysis (Supplementary Figure S1), which revealed that biological samples could be differentiated according to treatment (*M. bovis*-infected or non-infected samples) on dimension 1 and 2 starting at 6 hpi, with further discrimination at later time points.

**Figure 1:**
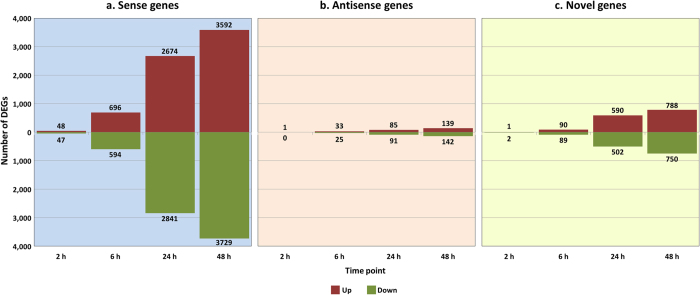
Number of significant DE genes at each time point post-infection. The numbers of upregulated and downregulated genes, in the *M. bovis*-infected alveolar macrophages relative to the control non-infected cells at each time point, are shown for (**a**) sense, (**b**) antisense and (**c**) novel gene analyses (adjusted *P*-value ≤ 0.05).

Statistical analysis identified 95 (48 up-, 47 downregulated), 1,290 (696 up-, 594 downregulated), 5,515 (2,674 up-, 2,841 downregulated), and 7,321 (3,592 up-, 3,729 downregulated) significantly DE genes (FDR-adjusted *P* ≤ 0.05) in the *M. bovis*-infected macrophages relative to the control non-infected macrophages at 2, 6, 24 and 48 hpi, respectively (Fig. 1a and Supplementary Data S3, worksheet 1). Among the top significantly upregulated sense genes were *HCAR3*, *TNFAIP6*, *TNFAIP3*, *MAFF* and *NFKBIZ*; while the top significantly downregulated sense genes were *DUSP7*, *RNF169*, *EZH1*, *IL6R* and *CDC42ER3* (ranked by the average of FDR-adjusted *P* value across all time points). This increase in the number of DE genes over the alveolar macrophage infection time course has been reported in other studies using bovine MDM^13^ and human alveolar macrophages^51^; these studies also observed higher fold-changes in gene expression for upregulated compared to downregulated genes.

The use of a strand-specific RNA-seq library preparation protocol also facilitated detection and quantification of transcripts that were located on the antisense DNA strand corresponding to an annotated sense gene. Natural antisense transcripts (NATs) have previously been reported in mammals with a wide range of functions, including transcriptional and post-transcriptional gene expression regulation, splicing event control and the regulation of allele-specific transcription^52^,^53^,^54^. Differential expression analyses of putative NATs involved both low expression and antisense ratio filtering criteria, which yielded 565 NATs suitable for downstream analyses. Statistical analysis identified 1 (1 upregulated), 58 (33 up-, 25 downregulated), 176 (85 up-, 91 downregulated), and 281 (139 up-, 142 downregulated) significantly DE NATs (FDR-adjusted *P* ≤ 0.05) in the *M. bovis*-infected relative to the control non-infected alveolar macrophages at 2, 6, 24 and 48 hpi, respectively (Fig. 1b and Supplementary Data S3, worksheet 2).

Finally, RNA-seq was used to identify putative bovine novel genes and refine the incomplete *B. taurus* genome annotation. Differential expression analyses of novel genes—transcription occurring in intergenic location of the reference gene annotation as identified via *de novo* transcriptome reconstruction^38^—also involved a low expression filtering step and yielded 3,088 putative novel genes suitable for analysis. Statistical analysis identified 3 (1 up-, 2 downregulated), 179 (90 up-, 89 downregulated), 1,092 (590 up-, 502 downregulated), and 1,538 (788 up-, 750 downregulated) significantly DE putative novel genes (FDR-adjusted *P* ≤ 0.05) in the *M. bovis*-infected relative to the control non-infected alveolar macrophages at 2, 6, 24 and 48 hpi, respectively (Fig. 1c and Supplementary Data S3, worksheet 3). It is important to note that further analysis described below led to the removal of a large number of artefactual putative novel genes.

### Correlation of sense and antisense gene expression

The DE NATs were compared to the DE sense genes according to direction of expression for each time point post-infection. In total, 1, 20, 114, and 195 genes showed the same direction of expression (*i.e*., up- or downregulated) on both the sense and the antisense strands at the 2, 6, 24 and 48 hpi time points, respectively. The *ACPP*, *LOC782264*, *GAS1*, *MBP*, *MSC* and *RERE* genes were among the top-ranking genes (based on NATs analysis FDR-adjusted *P* values), showing the same direction of expression for both sense genes and NATs at the 6, 24 and 48 hpi (Supplementary Figure S2). Furthermore, these genes have been shown to play a role in host defence mechanisms including immune development, resistance/susceptibility to infection and apoptosis^55^,^56^,^57^,^58^,^59^,^60^. In addition, we detected 3 and 10 genes at the 24 and 48 hpi time points, respectively, that exhibited opposing directions of expression on the antisense and sense strands (*i.e*., the NAT is up- and the sense gene is downregulated, or *vice versa*). These genes included *ACAD10*, *RHOBTB3* and *SLC30A4*, which have been shown to mediate the response to infection including endocytosis, zinc homeostasis and bactericidal mechanisms^61^,^62^,^63^.

DE sense genes may be subject to regulation mediated by putative NATs, which carry out a large range of functions by acting in *cis* and/or *trans* through various transcriptional and post-transcriptional regulatory mechanisms^52^,^53^. Firstly, NATs can interact with DNA to induce chromatin remodelling via DNA methylation or histone-modifying enzyme recruitment, leading to (de)repression of sense transcription^64^; consequently, we hypothesise *MSC* gene expression is mediated via such a mechanism through its associated promoter-located NAT. Secondly, the interaction of a NAT with sense RNA can lead to the formation of an RNA duplex, which can result in sense RNA alternative splicing, change in sense RNA localisation, modification of sense RNA stability and formation of small interfering RNA^65^,^66^; therefore, the downregulation of the NAT for *MBP* may reduce the stability and abundance of its sense RNA. Thirdly, the process of NAT transcription can provoke transcriptional collision, where convergent transcription occurs on opposite strands resulting in collision of two RNA polymerases and aborted sense RNA transcription^67^; this suggests a regulatory mechanism whereby the NAT of *SLC30A4* represses the sense gene. Sense gene expression regulation, modulated by NATs, may constitute host-mediated control of the immune response to fine-tune and tightly regulate the levels of proinflammatory cytokines and bactericidal molecules induced during infection^65^,^66^. Alternatively, such NAT-mediated regulation may comprise an immune evasion mechanism evoked by *M. bovis* to enable its survival within the macrophage. Finally, due to debate in the literature about the existence of functional NATs in vertebrates^68^,^69^,^70^, we implemented a stringent NAT detection filtering step to exclude artefactual NATs.

### Annotation of novel genes

The *de novo* transcriptome analysis facilitated detection of novel bovine genes, which were subsequently examined for differential expression between *M. bovis*-infected and control non-infected alveolar macrophages. The RBH method was used to annotate putative novel genes based on *H. sapiens* protein reference sequences^39^. Of the 3,088 novel genes that passed the low expression filtering step, it was possible to annotate 103 novel genes by comparison to pre-existing *H. sapiens* orthologs (Supplementary Data S3, worksheet 3). This suggested that a large number of the putative novel genes we detected may be artefacts; therefore, we focused subsequently only on bovine novel genes with *H. sapiens* orthologs.

Irrespective of their differential expression, these annotated novel bovine genes are important for refining the *B. taurus* genome annotation. In the context of the present study, several annotated novel bovine genes may be of particular importance; for example, *LILRA5* (upregulated at 24 and 48 hpi) encodes a leukocyte immunoglobulin-like receptor that induces production of IL-10 and other proinflammatory cytokines in macrophages^71^. In addition, *KLF13* (downregulated at 6, 24 and 48 hpi) encodes a transcription factor that negatively regulates macrophage alternative activation (M2 polarisation); alternatively activated macrophages have diminished bactericidal activity but enhanced phagocytic activity^72^. Also, the *KLF2* gene (upregulated at 48 hpi) encodes a negative regulator of proinflammatory cytokines and it is significant that macrophage migration and adhesion is reduced by increased expression of *KLF2*^73^. In summary, *LILRA5*, *KLF13* and *KLF2* encode proteins with important immunoregulatory functions for macrophages upon infection (*e.g*., regulation of inflammation to avoid potential cell damage), but also enhance survival of intracellular bacteria (*e.g*., reduction of bactericidal activity and proinflammatory cytokine production).

### RT-qPCR validation

We performed technical validation of the RNA-seq results by quantifying a panel of eight immune genes via RT-qPCR^25^ using the same RNA samples that were used for the RNA-seq analysis. Of the panel of genes analysed using RT-qPCR, six were significantly upregulated (*CCL4*, *IL10*, *IL1B*, *IL6*, *TLR2* and *TNF*) and two were significantly downregulated (*FOS* and *PIK3IP1*) at one or more post-infection time points. The comparison of RT-qPCR and RNA-seq results yielded a concordance (in terms of both direction of fold-change and statistical significance) of 71.9% across all eight genes. Comparison of the mean log_2_ fold-change in gene expression between the RT-qPCR and RNA-seq results revealed an overall (across all genes) Pearson correlation (*r*) value of 0.98 (*P*-value ≤ 0.001); while six genes (*CCL4*, *FOS*, *IL1B*, *IL6*, *PIK3IP1* and *TNF*) had *r*-values ≥ 0.97 (*P*-value ≤ 0.05) and two genes (*IL10* and *TLR2*) had *r*-values between 0.92-0.94 (*P*-value ≤ 0.1). The RT-qPCR results for technical validation of the RNA-seq data are provided in Supplementary Data S1, worksheet 2.

RT-qPCR biological validation of the RNA-seq results was also performed using the same panel of eight genes. For this, total RNA was extracted and purified from *M. bovis*-infected and non-infected control alveolar macrophages obtained from four additional animals. A concordance (in terms of the direction of fold-change and statistical significance) of 56.3% and an *r*-value of 0.96 (*P*-value ≤ 0.001) was obtained between the RT-qPCR results and the RNA-seq data for the tested genes. In addition, we observed *r*-values ≥ 0.99 (*P*-value ≤ 0.01) for *CCL4*, *IL1B*, *IL6*, and *TLR2*, and an *r*-value of 0.93 (*P*-value ≤ 0.1) for *TNF*; while the other three genes (*FOS*, *IL10* and *PIK3IP1*) were not significant, presumably due to the smaller sample size used for biological validation. The RT-qPCR results for biological validation of the RNA-seq data are provided in Supplementary Data S1, worksheet 3.

### Function and pathway enrichment analyses

To gain a better understanding of the macrophage cellular pathways modulated following *M. bovis* infection, we performed canonical pathway analyses using the IPA (Supplementary Data S4), Pathway-Guide (Supplementary Data S5) and Sigora (Supplementary Data S6) software tools with the DE genes identified using RNA-seq at each post-infection time point. We considered only canonical pathways flagged as significant by at least two of these tools. For the most part, these cellular pathways are involved in macrophage recognition of pathogens and subsequent signalling cascades that culminate in the activation of innate and adaptive immune processes.

### Recognition of mycobacteria by host macrophages

Two of the enriched canonical pathways involved in the recognition of mycobacteria by host macrophages were *Toll-like receptor signalling* and *RIG-I-like receptor signalling*. Further inspection of these pathways revealed upregulation of Toll-like receptor (TLR) genes previously shown to have important roles in mycobacterial recognition and activation of immune responses^74^. These included *TLR2* (encodes TLR2, which recognises bacterial peptidoglycan, lipoprotein and lipoarabinomannan), *TLR3*, *TLR4* and *CD14* (encodes a co-receptor in lipopolysaccharide recognition), all of which were upregulated at 6, 24 and/or 48 hpi. Notably, the *MYD88* gene, which encodes a key adaptor protein that transduces intracellular TLR signalling, was downregulated at 24 and 48 hpi. However, all the upregulated TLR genes can engage the MYD88-independent signalling pathway via the alternative adapter molecules encoded by *TICAM1* (upregulated at 6, 24 and 48 hpi) and *TICAM2* (upregulated at 48 hpi). The transcriptional response we observe here represents *M. bovis*-mediated modulation of host immune responses to facilitate pathogen survival within the host macrophage. Indeed, earlier studies—using *M. tuberculosis*-infected macrophages—have shown that prolonged mycobacterial stimulation (24 to 48 h stimulation) of TLRs (particularly TLR2) gives rise to inhibition of several IFN-γ-induced immune responses, particularly the expression of MHC class II molecules, thereby repressing the antigen presentation process^75^,^76^. Such inhibition of IFN-γ-induced MHC class II expression has been shown to involve MYD88-dependent or -independent TLR signalling^77^. Conversely, the upregulation of both *TICAM1* and *TICAM2* may act to circumvent the suppression of MYD88-dependent TLR signalling by the host to activate the immune response to control infection.

**Figure 2:**
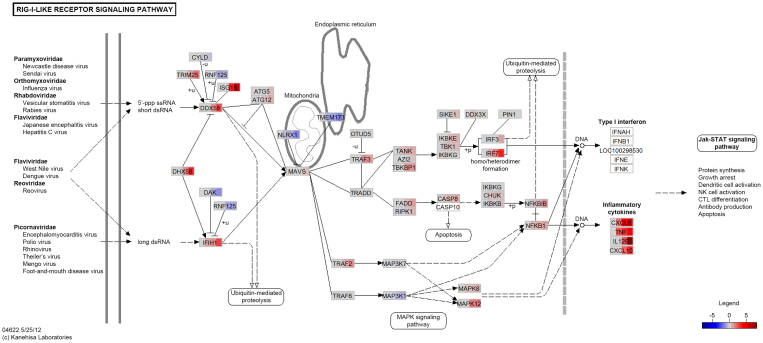
RIG-I-like receptor signalling pathway. The *RIG-I-like receptor signalling* pathway is represented with gene expression (log_2_ fold-change) values overlaid at all post-infection time points; shown from left to right are the 2, 6, 24 and 48 hpi time points, respectively. The colour intensity corresponds to the level of upregulation (red) or downregulation (blue) in the *M. bovis*-infected versus the control non-infected alveolar macrophages. Genes coloured in grey were not significantly DE and genes coloured in white were filtered out due to low expression.

We also observed that the RIG-I-like receptor (RLR) genes were upregulated at 6, 24 and/or 48 hpi; these were *DDX58* (encodes the RIG-I protein that recognises 5′ triphosphate single-stranded RNA and short double-stranded RNA), *IFIH1* (encodes a protein recognising long double stranded RNA) and *DHX58* (encodes a protein that positively and/or negatively regulates *DDX58* and *IFIH1*)^78^,^79^. Also, the *MAVS* gene, which encodes a key mitochondrial membrane-bound intermediary protein in the RLR signalling cascade, was upregulated at 48 hpi. In general, RLR signalling results in the activation of the IRF transcription factor complex (*IRF3* and *IRF7* were upregulated at 24 and/or 48 hpi), followed by type I interferons production that help control bacterial infection (*e.g*., enhancement of MHC class I antigen presentation)^80^,^81^ or can benefit bacterial survival (*e.g*., inhibition of the host immune response)^82^. Overall, the type I interferon-dependent immune response and outcome is largely pathogen- and tissue-specific; this is supported by the literature on models of *M. tuberculosis* infection^83^,^84^. In the current study, we did not observe expression of type I interferon genes and this could be due to a number of reasons. It is possible that alveolar macrophages respond to *M. bovis* infection by recruiting ATG5-ATG12 complex (genes upregulated at 24 and/or 48 hpi)—a key regulator of the autophagic process—that has been shown to interfere with RLR signalling^85^. Alternatively, *M. bovis* may actively inhibit induction of type I interferons, indeed *M. tuberculosis* can mediate such inhibition through TLR2 recognition^84^. Figure 2 shows the *RIG-I-like receptor signalling* pathway with gene expression values overlaid at all post-infection time points.

### Activation and recruitment of bactericidal immune mechanisms

The recognition and uptake of *M. bovis* by alveolar macrophages leads to induction/repression of several immune-related transcription factors (*e.g*., the NF-κB, STAT and IRF complexes) and culminates in the activation and recruitment of bactericidal immune processes. In the present study, the macrophage bactericidal immune mechanisms enriched in response to *M. bovis* infection were the *Apoptosis* and *Lysosome* canonical pathways. Inspection of these pathways revealed upregulation at 6, 24 and 48 hpi of *CASP8*, *CASP7*, *BID* and *CYCS*, which encode several pro-apoptotic proteins. However, a larger number of genes encoding inhibitors of apoptosis were upregulated at 2, 6, 24 and/or 48 hpi, such as *BCL2A1*, *CFLAR*, *BCL2*, *BCL2L1*, *BIRC2*, *BIRC3*, *XIAP*, *MCL1* and *PRKX*. Furthermore, nearly all genes encoding apoptotic endonucleases or effector molecules—which are key features for effective apoptosis—were downregulated at 24 and/or 48 h, including *ENDOG*, *DFFA* and *AIFM1*^86^. Several studies have reported reduced mycobacterial survival resulting from apoptotic cell death, which actively destroys the host cell and its contents, including intracellular bacteria such as *M. bovis*^86^,^87^. However, it is possible that virulent *M. bovis* actively represses host cell apoptotic processes *in vitro*, thereby facilitating persistence within host cells^88^,^89^; the upregulation of anti-apoptotic genes observed here supports this hypothesis.

**Figure 3:**
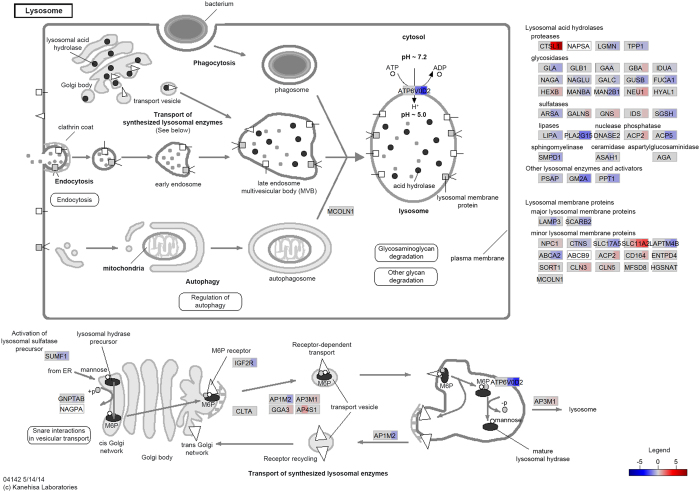
*Lysosome* pathway. The *Lysosome* pathway is represented with gene expression (log_2_ fold-change) values overlaid at all post-infection time points; shown from left to right are the 2, 6, 24 and 48 hpi time points, respectively. The colour intensity corresponds to the level of upregulation (red) or downregulation (blue) in the *M. bovis*-infected versus the control non-infected alveolar macrophages. Genes coloured in grey were not significantly DE and genes coloured in white were filtered out due to low expression.

We also observed downregulation at 6, 24 and/or 48 hpi of many genes encoding proteins involved in the transport and activation of lysosomal enzymes, such as *SUMF1*, *GNPTAB*, *IGF2R*, *AP3B1*, *AP4M1*, *GGA1* and the AP-1 assembly complex genes. Furthermore, the majority of genes encoding lysosomal enzymes were downregulated at 6, 24 and/or 48 hpi, notably the glycosidases, lipases, nucleases, sphingomyelinases, ceramidase, and prosaposin. Conversely, the genes encoding proteins mediating the acidification of lysosome compartments were mostly upregulated (*i.e*., *TCIRG1* and V-ATPase family members), with the exception of *ATP6V0D2* and *ATP6V0A1* (downregulated at 24 and 48 hpi). Interestingly, a gene encoding a key transcription factor of the *Lysosome* pathway, TFEB, was also downregulated at 6, 24 and 48 hpi^90^. Previous studies have characterised the repression of phagosome-lysosome fusion as a hallmark of infection by mycobacteria^91^,^92^. Our results also show suppression of lysosomal function by *M. bovis* via downregulation of a majority of key effector lysosomal enzymes. Figure 3 shows the *Lysosome* pathway with gene expression values overlaid.

### Comparison of the alveolar macrophage and monocyte-derived macrophage responses to tubercle bacilli

We next compared the transcriptomic responses for two different bovine host cell types in response to *M. bovis* infection: alveolar macrophages and monocyte-derived macrophages (MDM). RNA-seq expression data (accession number GSE45439) from *M. bovis*-infected and non-infected MDM were used from a study previously published by our group^14^. This experiment consisted of a single time point contrast (24 hpi), which was therefore compared to the contrast between *M. bovis*-infected and non-infected alveolar macrophages at 24 hpi.

The correlation between gene expression fold-changes (*M. bovis*-infected versus non-infected control groups) for the alveolar macrophage and MDM at 24 hpi was investigated for all genes that passed the low expression filtering in both experiments (number of genes = 10,314). A Spearman rank correlation coefficient of 0.352 (*P*-value ≤ 1 × 10^−15^) was observed, indicating a moderate correlation of gene expression fold-changes between the two experiments. A much larger number of DE genes (FDR adjusted *P*-value ≤ 0.05) were detected for the alveolar macrophage contrast at 24 hpi (2,674 up-, 2,841 downregulated) compared to the MDM experiment (1,392 up-, 1,192 downregulated). Of these DE genes, 1,220 were identical and exhibited the same direction of expression in both experiments (638 up-, 582 downregulated). Conversely, 240 genes were DE in both experiments but displayed an opposite direction of expression (Supplementary Figure S3). The concordant genes (1,220) between both experiments represented 22% of the total number of DE genes for the alveolar macrophage and 47% for the MDM. We also detected 307 and 195 enriched canonical pathways at 24 hpi for the alveolar macrophage and MDM, respectively; of these enriched canonical pathways, 52 were common to both cell types. Taken together, these findings demonstrate that primary alveolar macrophages, as the target host cell, provide a better model for studies of infection with tubercle bacilli. Notwithstanding this, the canonical pathways that overlapped between the two cell types related to pattern recognition receptors, NF-κB activation, apoptosis and cytokine-mediated cell signalling, suggesting that MDM can also provide meaningful, but arguably more limited, immunobiological information if used for *in vitro* studies of mycobacterial infection. Other groups have reported similar gene expression results for comparisons between alveolar macrophages and MDM infected with *M. tuberculosis* or stimulated with lipopolysaccharides^93^,^94^.

It is important to highlight a number of experimental factors that may have also contributed to the differences observed between the two studies, including: (1) the number of biological replicates (*n* = 10 for the present study versus *n* = 6 for the MDM); (2) the age and sex of the animals (young male calves versus mature four year-old females); (3) the bacterial culture media (Tween^−^ versus Tween^+^); (4) the MOI (10:1 versus 2:1); (5) differences in the RNA-seq library protocols used; (6) the high-throughput sequencing apparatus (Illumina^®^ HiSeq^™^ 2000 versus Genome Analyzer IIx); and (7) differences in the computational pipelines used for analysis of the RNA-seq data.

In conclusion, we have, for the first time, applied RNA-seq technology to define the response of the mammalian host alveolar macrophage to infection with tubercle bacilli. Our results with *M. bovis* and the bovine alveolar macrophage have clear parallels to *M. tuberculosis* and tuberculosis disease in humans. Our analysis highlights the complex gene expression patterns and regulation underlying the early mammalian host response during infection with tubercle bacilli. Notably, we report the involvement of NATs in the innate immune response to intracellular bacilli, which may play a previously underappreciated role in the pathogen-mediated suppression of host defence mechanisms. Furthermore, we have dissected the innate immune response to *M. bovis*-infection in unprecedented detail through identification and inclusion of previously unannotated novel bovine genes. Our results show involvement of Toll-like and RIG-I-like receptors, apoptosis and lysosome signalling, as well as repression of these mechanisms that indicates subversion of protective host processes by *M. bovis* to promote its survival and persistence within the macrophage and ultimately establish infection.

## Additional Information

**How to cite this article**: Nalpas, N. C. *et al*. RNA sequencing provides exquisite insight into the manipulation of the alveolar macrophage by tubercle bacilli. *Sci. Rep*. **5**, 13629; doi: 10.1038/srep13629 (2015).

## Supplementary Material

Supplementary Information

Supplementary Data S1

Supplementary Data S2

Supplementary Data S3

Supplementary Data S4

Supplementary Data S5

Supplementary Data S6
