# Supplementary material for: RNA sequencing provides exquisite insight into the manipulation of the alveolar macrophage by tubercle bacilli
Source: Sci Rep. 2015 Sep 8;5:13629. doi: 10.1038/srep13629 (PMC4642568; doi:10.1038/srep13629)
Supplement: Supplementary Information [file srep13629-s1.doc]

**RNA sequencing provides exquisite insight into the manipulation of the alveolar macrophage by tubercle bacilli**

Nicolas C. Nalpas, David A. Magee, Kevin M. Conlon, John A. Browne, Claire Healy, Kirsten E. McLoughlin, Kévin Rue-Albrecht, Paul A. McGettigan, Kate E. Killick, Eamonn Gormley, Stephen V. Gordon and David E. MacHugh^*^

* Corresponding author: David E. MacHugh

Corresponding author email address:

David E. MacHugh [david.machugh@ucd.ie](mailto:david.machugh@ucd.ie)

Supplementary information

**Supplementary Methods.** Detailed materials and methods.

**Supplementary Figure S1.** Multidimensional scaling plots of *M. bovis*-infected and non-infected samples based on RNA-seq sense gene data.

Dimension 1 and 2 separate *M. bovis*-infected and control samples at each time point post-infection based on the expression values of the 11,928 filtered sense genes. The *M. bovis*-infected and control non-infected samples are coloured in blue and red, respectively.

**Supplementary Figure S2.** Venn diagrams showing differential gene expression comparisons between sense and antisense genes.

**Supplementary Figure S3.** A Venn diagram showing the sense gene expression comparison for the separate alveolar macrophage and MDM experiments at 24 h post-infection.

Supplementary Methods

The laboratory methods used to isolate, culture and infect alveolar macrophages have previously been described by us[^1^](#_ENREF_1). The complete bioinformatics pipeline used for the computational and bioinformatics analyses, consisting of Perl/Unix Bash/R environment scripts, is accessible online[^2^](#_ENREF_2).

Ethics statement

All animal procedures were performed according to the provisions of the Cruelty to Animals Act of 1876 and ethical approval for the study was obtained from the University College Dublin (UCD) Animal Ethics Committee (protocol number AREC-13-14-Gordon).

Animal work, lung lavages and lung cell isolation

In the current study, fourteen unrelated, age-matched Holstein-Friesian male calves (7-12 weeks old) were selected from a tuberculosis-free, screened annually using the single intradermal comparative tuberculin skin test herd and maintained under uniform housing conditions and nutritional regimens at the UCD Lyons Research Farm (Newcastle, County Kildare, Ireland).

Total lung cells were harvested by pulmonary lavage of lungs obtained post-mortem. All lungs were visually inspected by a veterinary surgeon for signs of inflammation and pathology before proceeding with lung washes. Lungs were washed in a laminar flow hood. For this, sterile calcium- and magnesium-free Hank's Balanced Salt Solution (HBSS) [Invitrogen™, Life Technologies Corp., Paisley, UK] was infused into the lungs (500 ml per infusion; repeated approximately six times) via the trachea. Lungs were gently massaged and the HBSS-cell suspension was collected into sterile beakers. A 50 ml aliquot of HBSS-cell suspension collected from the first 500 ml HBSS infusion was centrifuged at 200 × g for 10 min at room temperature (RT) and the resulting cell pellet was resuspended in 10 ml HBSS. This 10 ml HBSS-cell suspension was screened for microbial contamination by incubation on agar plates using the following conditions: Columbia blood agar with 5% defibrinated sheep blood (aerobic and CO_2_-enriched atmosphere, 37 °C, 36 h); Chocolate agar (CO_2_-enriched atmosphere, 37 °C, 36 h); Columbia-colistin-nalidixic acid agar (aerobic, 37 °C, 36 h); MacConkey agar number 2 (aerobic, 37 °C, 36 h); Sabouraud dextrose agar (aerobic, 37 °C, 5 days); and Mycoplasma agar (CO_2_-enriched atmosphere, 14 days). All media was obtained from Oxoid Ltd. (Basingstoke, Hampshire, UK). All animals tested negative for microbial contamination in the lungs.

The remaining HBSS-cell suspension (approximately 1.5 l) was transferred to 50 ml sterile tubes and centrifuged (200 × g for 10 min at RT). The resulting cell pellets were pooled and resuspended in 50 ml cold R10^−^ media (RPMI 1640 medium [Invitrogen™, Life Technologies Corp.] supplemented with 10% foetal bovine serum (FBS), 2.5 µg/ml amphotericin B, and 2 mM L-glutamine [all sourced from Sigma-Aldrich Ltd., Dublin, Ireland]). The cell suspension was centrifuged (200 × g for 10 min at RT) and resuspended in 10 ml R10^+^ media (R10^−^ media supplemented with 100 μg/ml ampicillin and 25 μg/ml gentamycin [all sourced from Sigma-Aldrich Ltd.]). Cells were then counted using a haemocytometer, centrifuged (200 × g for 10 min at RT) and resuspended in 90% FBS with 10% dimethyl sulfoxide (Sigma-Aldrich Ltd.) at a density of 2.5 × 10^7^ cells/ml. Cells were aliquoted (1 ml per aliquot) in 2 ml sterile cryovials (Sarstedt Ltd., Wexford, Ireland) and placed into Mr. Frosty® Cryo 1 °C Freezing Containers (Nalgene®, Thermo Fisher Scientific, Waltham, MA, USA) containing 100% isopropyl alcohol. Cryovials were stored at −80 °C for a period of 20 h after which they were removed from the freezing containers and transferred to −140 °C freezer storage conditions until required for further use.

Alveolar macrophage culture, purification and flow cytometry

Approximately six vials of total lung cells from each animal (representing 1.5 × 10^8^ total lung cells) were removed from −140 °C storage and thawed by placing in a 37 °C water bath for 1 min. Once thawed, cells from the same animal were immediately transferred into 20 ml pre-warmed R10^+^ media (note that all pre-warmed media and solutions were heated to 37 °C prior to use) and centrifuged (200 × g for 5 min at RT). The cell pellet was resuspended in 15 ml of R10^+^ media, placed in a 75 cm^2^ vented culture flask (CELLSTAR®, Greiner Bio-One Ltd., Stonehouse, UK) and incubated for 24 h at 37 °C, 5% CO_2_. After incubation, media was removed together with non-adherent cells, and adherent cells were washed with 15 ml pre-warmed HBSS. After removing HBSS, adherent cells were dissociated by adding 10 ml pre-warmed cell dissociation solution non-enzymatic 1× (Sigma-Aldrich Ltd.) per culture flask and incubating at RT for 10 min. Dissociated cells were transferred to 30 ml of pre-warmed R10^+^ media, centrifuged (200 × g for 5 min at RT), resuspended in 10 ml pre-warmed R10^+^ media and the number of viable cells was counted using a Beckman Coulter® Vi-CELL™ XR Cell Viability Analyzer and reagent kit (Beckman Coulter Inc., Hugh Wycombe, UK). Mean viable cell recovery was estimated at ≥ 80% for each animal. Cell concentrations for each animal were adjusted to 5 × 10^5^ viable cells/ml using pre-warmed R10^+^ media, seeded at 1 ml/well (5 × 10^5^ cells/well) in 24-flat well tissue culture plates (Sarstedt Ltd.) and incubated for 24 h at 37 °C, 5% CO_2_, until required for infection.

Prior to performing the *in vitro* infections, the identity and purity of the seeded alveolar cells were confirmed by flow cytometry (Conway Institute, UCD, Ireland). For this, cells from two adjacent wells on a tissue culture plate from the same animal were washed with pre-warmed HBSS and dissociated by adding 250 μl pre-warmed cell dissociation solution to each well and pooling the dissociated cells. The pooled dissociated cells were added to 1 ml pre-warmed R10^+^ media. Cells were centrifuged at 200 × g for 5 min at RT and the resulting cell pellet was resuspended in 600 μl of flow cytometry buffer (sterile Phosphate-Buffered Saline [Invitrogen™, Life Technologies Corp.] supplemented with 0.1% Foetal Bovine Serum and 0.1% sodium azide [Sigma-Aldrich Ltd.]). The cells in one aliquot were stained with 0.25 μg mouse anti-bovine CD14 fluorescein-labelled IgG (Kingfisher Biotech Inc., St. Paul, MN, USA) for 30 min at RT followed by staining with 0.67 μg Alexa Fluor® 488 anti-mouse IgG antibody for 30 min at RT. The cells in the remaining aliquot were stained with 0.67 μg Alexa Fluor® 488 anti-mouse IgG antibody (Invitrogen™, Life Technologies Corp.) for 30 min at RT to detect and quantify non-specific cell-secondary antibody binding. All antibody preparations were made using flow cytometry buffer and all antibody incubation steps were performed in the dark. Cells were fixed by centrifuging the stained cells at 200 × g for 5 min at RT and resuspending the pellet in 200 μl 4% paraformaldehyde solution (Sigma-Aldrich Ltd.). Fixed cells were analysed on a FC500 flow cytometer (Beckman Coulter Inc., Fullerton, CA, USA) in the Conway Flow Cytometry Core (Conway Institute, UCD, Ireland). Analysis of flow cytometry raw data was performed within the R statistical programming environment (version 3.0.1)[^3^](#_ENREF_3) using the flowCore (version 1.26.2)[^4^](#_ENREF_4) and flowViz (version 1.24.0)[^5^](#_ENREF_5) Bioconductor packages. A gating strategy was performed to estimate the percentage of CD14^+^ alveolar macrophages in each animal sample. The estimated purity of alveolar macrophages for each animal sample was ≥ 95% (see pipeline accessible online[^2^](#_ENREF_2)).

Culture of *M. bovis*

Culturing of *M. bovis* AF2122/97 was performed in a Biosafety Level 3 laboratory and conformed to Irish national guidelines on the use of Hazard Group 3 infectious organisms. *M. bovis* AF2122/97 strain was obtained from the Animal Health and Veterinary Laboratory Agencies (AHVLA), Addlestone, Surrey, UK and was cultured to late logarithmic phase in 200 ml Middlebrook 7H9 media (Difco™, Becton, Dickinson Ltd., Oxford, UK) enriched with 1× Middlebrook albumin-dextrose-catalase (ADC) [Difco™] and 10 mM final concentration of sodium pyruvate (Sigma-Aldrich Ltd.). The *M. bovis* culture stocks were aliquoted (1 ml per aliquot) into sterile 2 ml cryovial tubes (Thermo Fisher Scientific Inc., Waltham, MA, USA) and stored at −80 °C until required.

Approximately 14 days prior to alveolar macrophage infection, 1 ml *M. bovis* culture stocks were removed from −80 °C storage, thawed, added to 4 ml Middlebrook 7H9-ADC medium containing 10 mM sodium pyruvate and cultured at 37 °C, 5% CO_2_ until mid-logarithmic phase (approximately 5-7 days). The 5 ml starter cultures were then transferred to 25 ml fresh Middlebrook 7H9-ADC medium containing 10 mM sodium pyruvate and cultured statically in vented 500ml Corning™ Erlenmeyer flasks (Thermo Fisher Scientific Inc.) at 37 °C, 5% CO_2_ until late-logarithmic phase (approximately 7 days).

On the day of alveolar macrophage infection, the 25 ml *M. bovis* culture was centrifuged (200 × g for 10 min); subsequently the cell pellet was disrupted by vortexing at top speed for 1 min in the presence of ten 3 mm sterile glass beads (Sigma-Aldrich Ltd.). Cells were resuspended in 5-10 ml pre-warmed R10^−^ media and left to stand at RT for 10 min to allow all cells to sediment, after which the uppermost 5 ml were transferred to a fresh sterile 50 ml tube and centrifuged at 200 × g for 10 min. Once centrifuged, the uppermost 4 ml were transferred to a fresh sterile 50 ml tube and sonicated at full power for 1 min in a Bransonic 2510 ultrasonic cleaner (Branson Ultrasonics Corp., Danbury, CT, USA). The OD_600nm_ of the culture was then measured and the number of cells was calculated based on an OD_600nm_ of 0.1 being equivalent to 1 × 10^7^ bacterial cells. The cell number was then adjusted to 5 × 10^6^ bacterial cells/ml using R10^−^ media.

*M. bovis*-infection of alveolar macrophages and macrophage RNA extraction

All *in vitro* infections were performed in a Biosafety Level 3 laboratory. For this, the R10^+^ media from all tissue culture plate wells containing alveolar macrophage (seeded at 5 × 10^5^ cells/well) was removed and replaced with 1 ml R10^−^ media containing *M. bovis* (5 × 10^6^ cells/ml), yielding a multiplicity of infection (MOI) of 10 bacilli per macrophage. Parallel non-infected control alveolar macrophage samples received 1 ml R10^−^ media only. All alveolar macrophage treatments (*i.e.* non-infected controls and *M. bovis*-infected samples) were performed in duplicate. Once infected, the alveolar macrophages were incubated at 37 °C, 5% CO_2_ for 2, 6, 24 and 48 h. After 2 h post-infection, the media from all 6, 24 and 48 h infection experiments was replaced with 1 ml/well of R10^−^ media and tissue culture plates were reincubated at 37 °C, 5% CO_2_ until the cells were required for harvesting. Infected and non-infected control alveolar macrophages were lysed by adding 250 μl/well RLT buffer supplemented with 1% β-mercaptoethanol (Qiagen Ltd., Crawley, UK). Alveolar macrophage lysates from duplicate treatments from the same animal were pooled and stored at −80 °C until required for RNA extraction.

All RNA extractions were performed using an RNeasy® Plus Mini kit (Qiagen Ltd.) according to the manufacturer’s instructions. RNA quantity and quality was assessed using a NanoDrop™ 1000 spectrophotometer (Thermo Fisher Scientific Inc.) and an Agilent 2100 Bioanalyzer with an RNA 6000 Nano kit (Agilent Technologies Ltd., Cork, Ireland). All samples displayed a concentration ranging from 10 up to 135 ng/μl (median 50 ng/μl), a 260/280 ratio greater than 2.0 and an RNA integrity numbers greater than 8.5. RNA samples were stored at −80 °C until required.

Strand-specific RNA-seq library preparation and sequencing

For the current study, 78 strand-specific RNA-seq libraries were prepared. These comprised *M. bovis*- and non-infected samples from each post-infection time points (2, 6, 24 and 48 h) across 10 animals (with the exception of one animal that did not yield sufficient alveolar macrophages for *in vitro* infection at the 48 h post-infection time point).

For RNA-seq library preparation, 200 ng of total RNA from each sample was used to prepare individually barcoded strand-specific RNA-seq libraries. Two rounds of poly(A)+ RNA purification were performed for all RNA samples using the Dynabeads® mRNA DIRECT™ Micro Kit (Invitrogen™, Life Technologies Corp.) according to the manufacturer’s instructions. The purified poly(A)+ RNA was then used to prepare individually barcoded strand-specific RNA-seq libraries using the ScriptSeq™ v2 RNA-Seq Library Preparation Kit, the ScriptSeq™ Index PCR Primers (Sets 1 to 4) and the FailSafe™ PCR enzyme system (all sourced from Epicentre®, Illumina® Inc., Madison, WI, USA) according to the manufacturer’s instructions. RNA-seq libraries were purified using the Agencourt® AMPure® XP system (Beckman Coulter Genomics, Danvers, MA, USA) according to the manufacturer’s instructions for double size selection (0.75× followed by 1.0× ratio). RNA-seq libraries were quantified using a Qubit® fluorometer and Qubit® dsDNA HS Assay Kit (Invitrogen™, Life Technologies Corp.), while library quality checks were performed using an Agilent 2100 Bioanalyzer and High Sensitivity DNA Kit (Agilent Technologies Ltd.). Individually barcoded RNA-seq libraries were pooled in equimolar quantities (10 nM of each individual library) and the quantity and quality of the final pooled libraries (three RNA-seq libraries pools in total) were assessed as described above.

Cluster generation and sequencing of the pooled RNA-seq libraries were performed by the Beijing Genomics Institute (BGI–Hong Kong, Hong Kong, China) using an Illumina® HiSeq™ 2000 sequencer. Each of the three pooled libraries was sequenced on eight lanes split across multiple Illumina® flow cells. The pooled libraries were sequenced as paired-end 2 × 90 nucleotide reads using Illumina® sequencing kits (version 5.0) and the standard Illumina® HiSeq™ 2000 pipeline. The Illumina® Sequencing Control Software (version 2.9) and Real Time Analysis (version 1.9) software packages were used for real-time tracking of the sequencing run, real-time image processing, the generation of base intensity values and base calling. All RNA-seq data generated for this study have been deposited in the NCBI GEO database with experiment series accession number GSE62506.

Bioinformatics analyses of RNA-seq data

Upon sequencing completion, computational analyses were performed on a 32-cores Linux Compute Server (4× AMD Opteron 6220 processors at 3.0 GHz with 8 cores each), with 256 GB of RAM (32× 8 GB at 1333 MHz), with 24 TB of hard disk drive storage (8× 3 TB at 7200 rpm) and with Linux Ubuntu as operating system (version 12.04.2). An initial quality check was performed on each of the raw reads data files using the FastQC software (version 0.10.1) to determine the most appropriate read cleaning strategy. Subsequently, a custom perl script was used to: (1) deconvolute the pooled libraries into individual libraries based on the unique index barcode (allowing up to one mismatch as long as the barcode sequence can be associated to a single unique index barcode); (2) filter out paired-end reads containing adapter sequence contamination (allowing up to three mismatches); and (3) remove paired-end reads of poor quality (*i.e.,* at least one of the reads containing 25% of bases with a Phred quality score below 20). The quality of the individual libraries files was then reassessed post-filtering using the FastQC software. Paired-end reads, from each filtered individual library, were aligned to the *B. taurus* reference genome (*B. taurus* UMD3.1.71 genome release; obtained from Ensembl at <ftp://ftp.ensembl.org/pub/release-71/fasta/bos_taurus/dna/>) using the STAR aligner software (version 2.3.0)[^8^](#_ENREF_8). A final quality check of the successfully aligned reads was performed with FastQC software to assess the sequence duplication level of each aligned individual library. In addition, paired-end reads of each filtered individual libraries were also aligned to the *M. bovis* AF2122/97 chromosome, complete genome (reference sequence NC_002945.3; obtained from NCBI at <http://www.ncbi.nlm.nih.gov/nuccore/31791177?report=fasta>) using STAR aligner software to determine the level of *M. bovis* sequence read contamination (see pipeline accessible online[^2^](#_ENREF_2)).

Sense genes data analyses

For each library, raw counts for each gene based on sense strand data were obtained using the featureCounts software from the Subread package (version 1.3.5-p4). The featureCounts parameters were set to unambiguously assign uniquely aligned paired-end reads in a stranded manner to the exons of genes within the *B. taurus* reference genome annotation from Ensembl (*B. taurus* UMD3.1.71 genome annotation; obtained from Ensembl at <ftp://ftp.ensembl.org/pub/release-71/gtf/bos_taurus/>)[^11^](#_ENREF_11). The gene count outputs were used to perform differential gene expression analysis using the edgeR Bioconductor package (version 3.2.4)[^12^](#_ENREF_12) within the R statistical programming environment. The differential gene expression pipeline within the edgeR package was customised to: (1) filter out all bovine rRNA genes; (2) filter out genes displaying expression levels below the minimally-set threshold of one count per million (CPM) in at least ten individual libraries (ten being the number of biological replicates per conditions); (3) calculate normalisation factors for each library using the trimmed mean of M-values method[^13^](#_ENREF_13); (4) generate the density of counts per gene and multidimensional scaling (MDS) plots based on data from each individual library; (5) estimate the dispersion parameter for each library using the Cox-Reid method; (6) identify differentially expressed genes between *M. bovis*-infected versus non-infected control samples from the same animal (*i.e.,* paired-sample statistical model) within each time point using a negative binomial generalized linear model; and (7) correct for multiple testing using the Benjamini-Hochberg method[^14^](#_ENREF_14) with a FDR threshold of 0.05 (see pipeline accessible online [^2^](#_ENREF_2)).

Antisense genes data analyses

For antisense gene analyses, the *B. taurus* reference genome annotation (*B. taurus* UMD3.1.71 genome annotation; obtained from Ensembl at <ftp://ftp.ensembl.org/pub/release-71/gtf/bos_taurus/>) was modified such that antisense genes are located on the opposite strand of their associated sense genes and to include a promoter (2,000 bases of promoter at 5' end) and terminator region (2,000 bases of terminator at 3' end)[^15-17^](#_ENREF_15). Furthermore, to limit false positive antisense detection, proximal genes closer than 5.0 kb were ignored for the custom *B. taurus* antisense genome annotation. For each library, a custom perl script was used to select uniquely aligned paired-end reads not previously assigned to any sense gene. The featureCounts parameters were set to unambiguously assign selected reads in a stranded manner to the full gene length (including promoter, exon, intron and terminator regions) of gene within the custom *B. taurus* antisense genome annotation.

To remove antisense gene artefacts, which may arise due to bias in the RNA-seq library preparation protocol[^18^](#_ENREF_18), we performed a gene counts ratio filtering procedure modified from a method described by Perocchi and colleagues[^19^](#_ENREF_19). For this step, the featureCounts software was used to generate the following for each library: (1) raw counts per antisense gene over exonic region only; and (2) raw counts per sense gene over the full gene length. Subsequently, for each library, the edgeR package was used to combine: (1) the sense gene raw counts over exonic region only (that has already been generated for the sense genes data analyses); (2) the antisense gene raw counts over exonic region only; (3) the sense gene raw counts over the full gene length; and (4) the antisense gene raw counts over the full gene length. Genes displaying expression levels below the minimally-set threshold of 20 CPM in at least ten individual libraries (the threshold was adapted to take into account the smaller library size of the antisense data compare to the sense data) were filtered out. A ratio of antisense counts over total counts was calculated based on exonic region only and based on the full gene length for each gene and individual library:


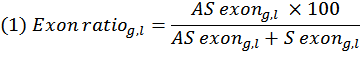


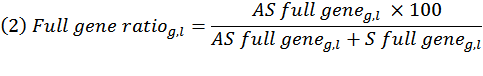


*AS* = antisense count; *S* = sense count; *g* = gene; *l* = library.

A greater-sided pairwise Wilcoxon signed-rank tests was then used to assess significant differences between the full gene ratio and the exon ratio for each gene. Antisense genes found to be significant with this ratio method were then used for differential gene expression analysis (between *M. bovis*-infected versus non-infected control samples for each time point) using the edgeR package and the raw counts (over full gene lengths) for each antisense gene according to the sense gene differential expression method described above (see pipeline accessible online[^2^](#_ENREF_2)).

Identification of novel bovine genes

For novel genes analyses, aligned reads were processed via the Cufflinks software package (version 2.1.1)[^20^](#_ENREF_20) using the reference annotation based transcript assembly method[^21^](#_ENREF_21) and the *B. taurus* reference genome annotation (*B. taurus* UMD3.1.71 genome annotation; obtained from Ensembl at <ftp://ftp.ensembl.org/pub/release-71/gtf/bos_taurus/>) to generate a *de novo* transcripts assembly. The *de novo* transcripts assembly files, obtained for each RNA-seq library, were merged into a single transcript assembly using the Cuffmerge script within the Cufflinks software package.

The reciprocal best hit (RBH) method was used to annotate putative novel genes. Nucleotide sequences for these putative novel genes were obtained using the gffread utility within the Cufflinks software package. Putative novel gene sequences were blasted against all *Homo sapiens* reference protein sequences (*H. sapiens* GRCh37.73 protein sequence; obtained from Ensembl at <ftp://ftp.ensembl.org/pub/release-73/fasta/homo_sapiens/pep/Homo_sapiens.GRCh37.73.pep.all.fa.gz>) using standalone BLAST® (version 2.2.28+)[^24^](#_ENREF_24). A custom perl script was then used to select the best *H. sapiens* protein hit for each *B. taurus* novel gene. Subsequently, the best *H. sapiens* protein sequence hits were reciprocally blasted against all *B. taurus* gene sequences (including reference genes and novel genes). Finally, a custom perl script was used to determine the RBH among the bovine novel genes identifed; RBH genes with a BLAST Expectation value (E-value) ≥ 1 × 10^−4^ were filtered out.

For each library, a custom perl script was used to select uniquely aligned paired-end reads not previously assigned to any sense or antisense gene. Following this, the featureCounts software was used to unambiguously assign selected reads in a stranded manner to exons for each gene ID from the *de novo* *B. taurus* genome annotation. For each library, the raw counts per novel gene were used for differential expression analysis (between *M. bovis*-infected versus non-infected control samples for each time point) using the edgeR package according to the previously described sense gene differential expression method. However, in this case the low expression filtering threshold was set to a minimum of 30 CPM in at least ten libraries—the threshold was adapted to take into account the much smaller library size of the novel data compare to the sense data (see pipeline accessible online[^2^](#_ENREF_2)).

Systems analyses

Using the biomaRt Bioconductor package (version 2.16.0)[^25^](#_ENREF_25), each *B. taurus* sense gene was annotated with its *H. sapiens* ortholog from Ensembl (*H. sapiens* GRCh37.71). We strictly kept *B. taurus* gene having a unique *H. sapiens* ortholog gene, and *vice versa*. This cross-annotation facilitated combining the differentially expressed sense genes with the differentially expressed novel genes data sets (all with *H. sapiens* Ensembl gene IDs), while also providing consistent data input for the different system biology analyses tools.

Ingenuity® Systems Pathway Analysis (IPA) software package (Qiagen Corp., Redwood City, CA, USA; release date December 2013) was used to identify over-represented biological functions and canonical pathways based on the Ingenuity® Knowledge Base. The Ingenuity® Knowledge Base contains the largest database of manually-curated and experimentally-validated physical, transcriptional and enzymatic molecular interactions, which are supported by previously published information. IPA performed an over-representation analysis that categorises differentially expressed genes into biological function groups and canonical pathways using the Ingenuity® Knowledge Base. The right-tailed Fisher’s exact test was used to calculate a *P*-value for each biological function and canonical pathways, and a multiple testing correction (Benjamini-Hochberg method) was applied with an FDR threshold ≤ 0.05.

To further focus on the canonical pathways of most relevance to our study, the Pathway−Guide software package (version 3.0.4; Advaita Corp., Plymouth, MI, USA) was used to perform signalling pathway impact analysis, which used biological interaction data from the KEGG[^28^](#_ENREF_28) and Reactome[^29^](#_ENREF_29) databases. Pathway-Guide calculated a global probability value for each pathway, incorporating parameters, such as the log_2_ fold-change of the differentially expressed genes, the statistical significance of the set of pathway genes and the topology of the signalling pathway. The over-representation analysis of the number of differentially expressed genes observed on the pathway is computed using a hypergeometric distribution. Then, the significance of observed total pathway perturbation was computed using a bootstrap procedure. Finally, the global probability value for each pathway was calculated by combining the pathway topology *P*-value with the pathway over-representation *P*-value, followed by correction for multiple testing (Benjamini-Hochberg method) was also applied with an FDR threshold ≤ 0.05.

Finally, using the R statistical programming environment, the Sigora package (version 0.9.2)[^30^](#_ENREF_30) was used to perform signature over-representation analysis based on the KEGG and Reactome databases. The Sigora package uses a novel approach to pathway analysis by identifying statistically over-represented pathway gene-pair signatures (*i.e.,* weighted pairs of genes which uniquely occur together in a single pathway). By focusing on unique features (called “Signatures”) of pathways, this approach accounts for the overlapping structure of pathway annotation. Sigora package assigned a weight to each gene-pair signature to quantify its reliability as an indicator of its associated pathway, followed by hypergeometric distribution of gene-pair signature weights to assess significance of pathways[^30^](#_ENREF_30). In this case, the Bonferroni method[^31^](#_ENREF_31) was used for multiple testing correction (see pipeline accessible online [^2^](#_ENREF_2)).

cDNA synthesis and RT-qPCR analysis

Technical and biological validation of the RNA-seq results was performed using reverse transcriptase quantitative real-time PCR (RT-qPCR). For technical validation, cDNA was prepared from the same RNA used for the RNA-seq library preparations. On the other hand, for biological validation, cDNA was prepared from RNA extracted from *M. bovis*- and non-infected alveolar macrophage samples obtained from four additional calves not used for the RNA-seq study. For both technical and biological validation analyses, cDNA was prepared from 60 ng of total RNA using a High Capacity cDNA Reverse Transcription Kit (Applied Biosystems®, Life Technologies Corp., Warrington, UK). cDNA conversions were performed in 20 µl reaction using random primers and MultiScribe™ Reverse Transcriptase (50 U per reaction) according to the manufacturer’s instructions. Reactions were incubated at 25 °C for 10 min, 37 °C for 2 h and 4 °C for 5 min. In addition, 60 ng of pooled RNA samples (approximately 10 RNA samples per pool) were used to generate non-reverse transcriptase (non-RT) control samples. All individual cDNA samples and non-RT controls were diluted 1:6 using RNAse- and DNAse-free water and were stored at −20 °C prior to RT-qPCR analysis.

Intron-spanning RT-qPCR primers (**Supplementary Data S1, worksheet 1**) were designed for each gene using the Primer3Plus software package—that included several *in silico* assessment for specificity (using NCBI-BLAST® software package[^24^](#_ENREF_24)), primers secondary structure formation (using Vector NTI® software [Invitrogen™, Life Technologies Corp.]) and RT-qPCR amplicon secondary structure formation (using RTprimerDB software package[^32^](#_ENREF_32))—and commercially synthesised (Eurofins MWG Operon, Ebersberg, Germany). RT-qPCR reactions (20 μl final volume) were performed on 96-well plates using Fast SYBR® Green Master Mix (Applied Biosystems®, Life Technologies Corp.) on a 7500 Fast Real-Time PCR System (Applied Biosystems®, Life Technologies Corp.) according to the manufacturer’s instructions. Reactions contained 3 μl diluted cDNA samples (corresponding to an estimated 1.5 ng cDNA), 10 μl of SYBR mix and 300 nM final concentration of each forward and reverse primer (**Supplementary Data S1, worksheet 1**). The appropriate non-RT and non-template controls were included to test for the presence of genomic DNA contamination, reagents contamination and primer-dimer formation. PCR thermal cycling conditions comprised one cycle at 50 °C for 2 min, one cycle at 95 °C for 20 s, followed by 40 cycles at 95 °C for 3 s and 60 °C for 30 s. A dissociation step was also included to confirm the presence of single discrete PCR products of the expected size; this was further confirmed by visualisation of the expected size amplification products on 2% agarose gels stained with 0.5 µg/ml ethidium bromide (Invitrogen™, Life Technologies Corp.).

RT-qPCR normalisation was performed using the qbase^+^ software package (version 2.6; Biogazelle NV, Zwijnaarde, Belgium)[^33^](#_ENREF_33). Normalisation of gene expression data was performed using two reference genes, *PPIA* and *H3F3A*, which were identified from a panel of eight candidate reference genes using the GeNorm algorithm[^34^](#_ENREF_34) within qbase^+^ software package. Calibrated normalised relative quantities (CNRQ) of gene expression obtained for each samples were transformed to log_2_ space and fold-change in gene expression was calculated by subtracting the log_2_ CNRQ of non-infected control samples from the log_2_ CNRQ of *M. bovis*-infected samples for the same animal at the corresponding time point. The SPSS statistical package (version 20.0.0; IBM Corp., Armonk, NY, USA) was used for statistical analyses of RT-qPCR data. The Shapiro-Wilk test was applied to the log_2_ fold-change in expression for each gene and time point to ensure normal distribution of data. Two-tailed paired sample *t*-tests and pairwise Wilcoxon signed-rank tests were used to assess significant differences in mean log_2_ fold-changes in gene expression (between the *M. bovis*-infected and the non-infected control samples at each time point) for normally and non-normally distributed data (Shapiro-Wilk test *P*-value ≤ 0.1), respectively. The R statistical programming environment was used to estimate the Pearson’s correlation of the mean log_2_ fold-change in gene expression between RT-qPCR and RNA-seq results.

Note: the RT-qPCR material and methods information provided above comply with the MIQE guidelines[^35^](#_ENREF_35).

References

Supplementary Figure S1

**
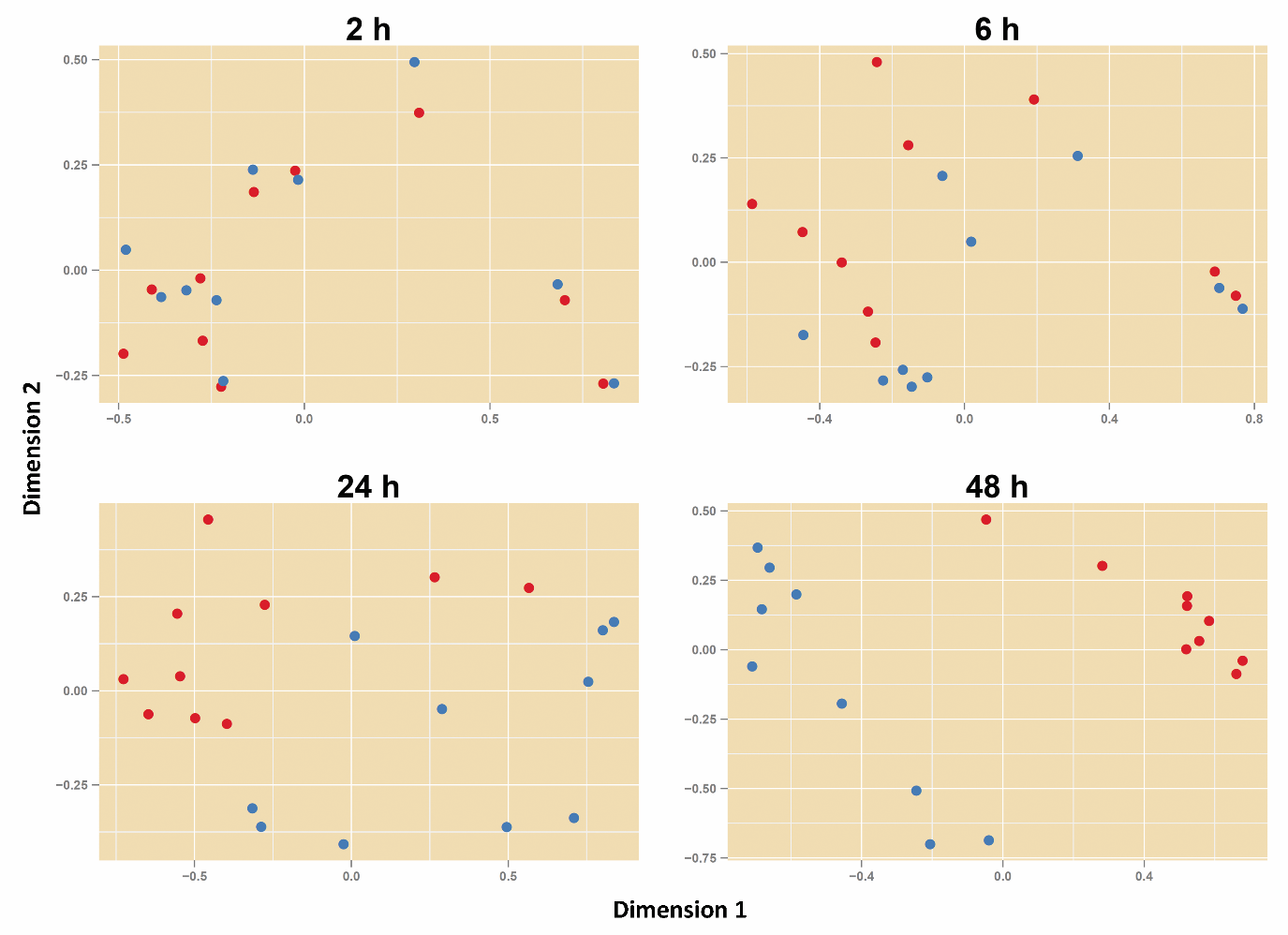
**

Supplementary Figure S2

**
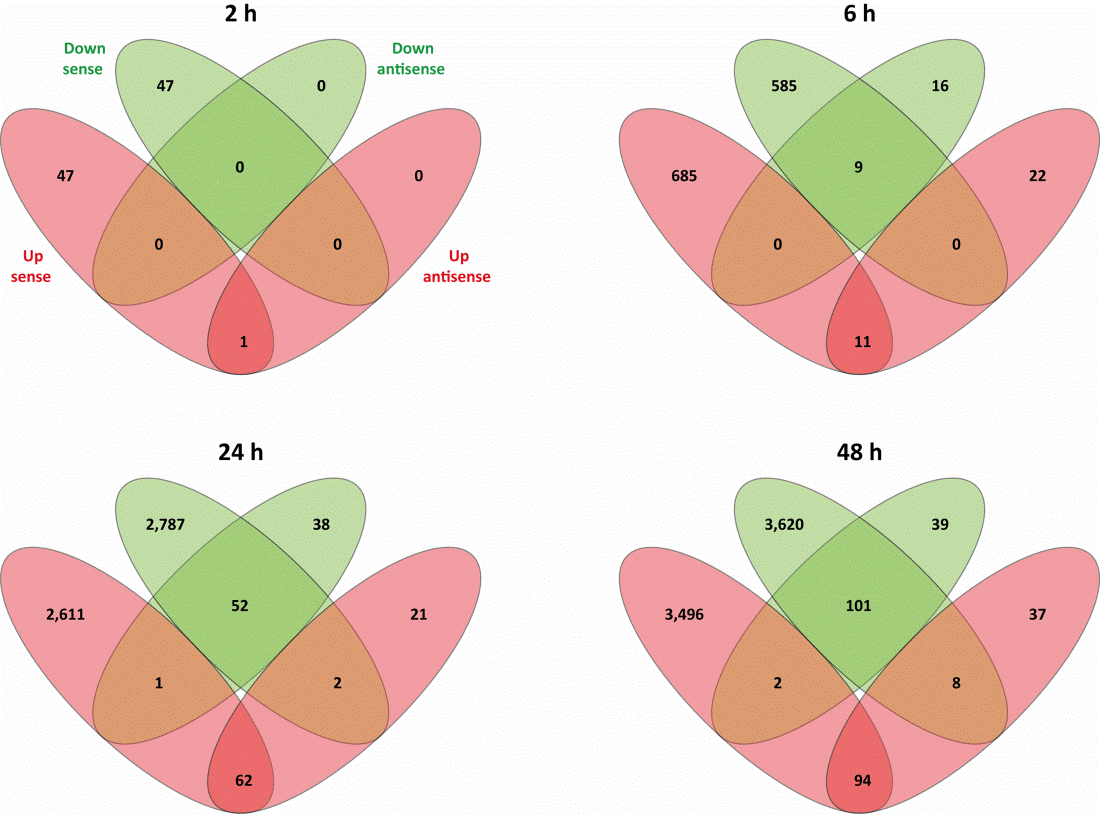
**

Supplementary Figure S3

**
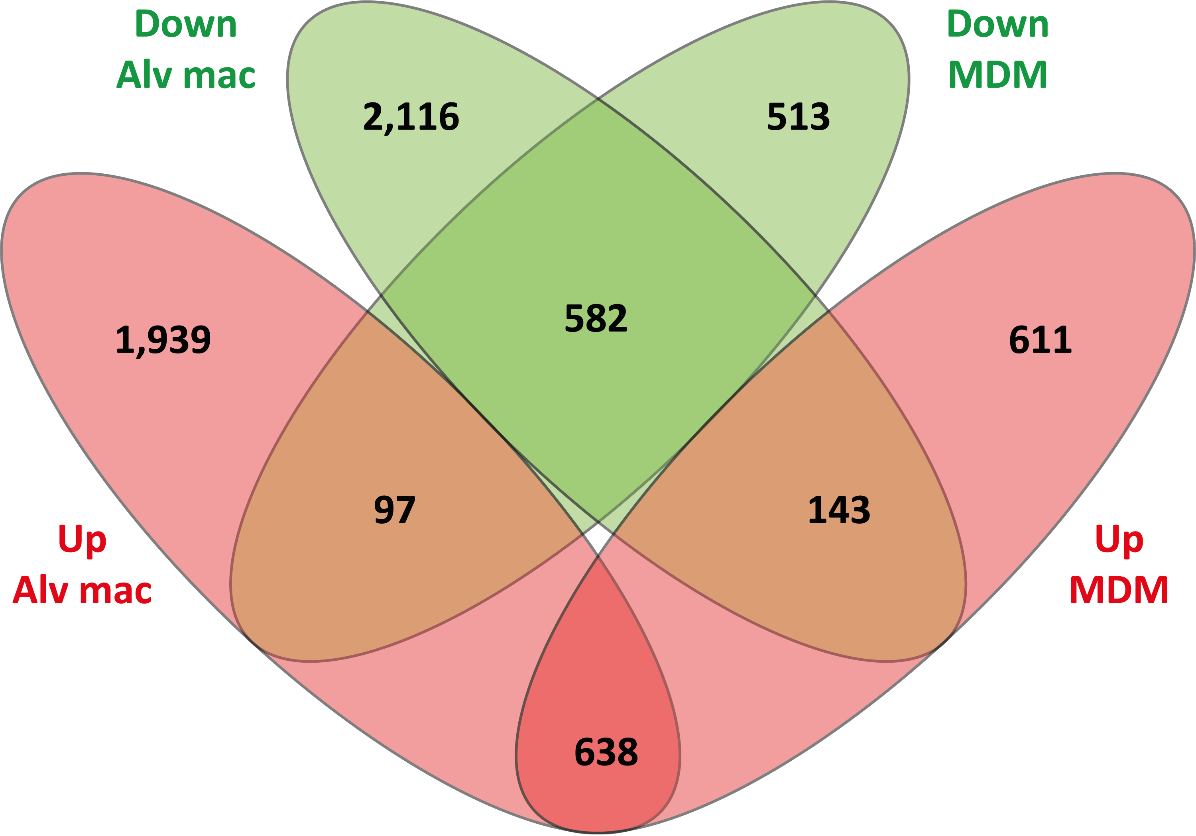
**
